# 50 The spectrum of pediatric lupus: data from the Kenya Pediatric Rheumatology Registry (KAPRI)

**DOI:** 10.1093/rheumatology/keac496.046

**Published:** 2022-10-07

**Authors:** A Migowa, R Odhiambo, J Orwa

**Affiliations:** 1 Aga Khan University Medical College East Africa, Department of Pediatrics and Child Health; 2 Aga Khan University Medical College East Africa, Centre of Excellence of Women and Child Health; 3 Aga Khan University Medical College East Africa, Department of Population Health

## Abstract

**Background:**

Pediatric rheumatic diseases are chronic illnesses that impart a significant disease burden upon children and their families (1–3). Furthermore, they have the potential to cause physical disability, diminished quality of life and significant direct and indirect costs (1–3). Determination of the burden and clinical characteristics of pediatric lupus is a critical first step to improving access to care and optimizing use of existing health systems for the well-being of these patients (4). Childhood-onset systemic lupus erythematosus (cSLE) is a prototype autoimmune condition characterized by simultaneous or sequential organ and system involvement, with unpredictable flare and high levels of morbidity and mortality (5). A pediatric rheumatology registry is critical in defining the spectrum of pediatric lupus within the region, the clinical characteristics of the disease, the outcomes and responses to various interventions. The Kenya Pediatric Rheumatology Registry (KAPRI) registry offers a unique opportunity to pioneer and spearhead a systematic and organized format in collecting pertinent clinical information that will offer information on current clinical scenarios and offer a platform to conduct other research projects to improve pediatric lupus healthcare in sub-Sahara Africa. Our objective was to determine the clinic-epidemiological profile of paediatric lupus patients by describing their baseline patient characteristics and clinical features at the Aga Khan University Medical College East Africa who were enrolled into the KAPRI registry from inception in March 2019 to December 2021.

**Methods:**

All patient records were selected from the KAPRI registry database using the ICD 10 code M32 that denotes Systemic Lupus Erythematosus (SLE) Age, gender, laboratory, clinical features at diagnosis, treatment options offered at time of diagnosis were extracted from the database.

**Results:**

Among the 207 patients enrolled thus far in the registry, 7 had a diagnosis of cSLE (3.4%). The commonest symptom among the patients were joint pain, fever and rash. Other clinical features and outcomes are highlighted in the table 1 below;

**Conclusion:**

Over a 2-year period, 57% of our cSLE patients (4 of 7) achieved remission while 43% (3of 7) were lost to follow up. Further studies are required to elucidate predictors of good clinical response and reasons for loss to follow up among our cSLE patients.

**References**

1. Brunner, H., Huggins J and Klein-Gitelman MS. Pediatric SLE—towards a comprehensive Management plan Nat. Rev. Rheumatol. 7, 225–233 (2011).

2. Kamphuis, S. and Silverman, E. D. Prevalence and burden of pediatric-onset systemic lupus erythematosus Nat. Rev. Rheumatol. 6, 538–546 (2010).

3. Migowa A, Colmegna I, Hitchon C, Were E, Ng’ang’a E, Ngwiri T, *et al.* The spectrum of rheumatic in-patient diagnoses at a pediatric hospital in Kenya. Pediatric Rheumatology (2017)

4. Trindade, V.C., Carneiro-Sampaio, M., Bonfa, E. *et al.* An Update on the Management of Childhood-Onset Systemic Lupus Erythematosus. Pediatr Drugs 23, 331–347 (2021).

5. Scott C, Webb K. Pediatric rheumatology in sub-Saharan Africa. Rheumatology (Oxford). 2014; 53(8):1357–8.

**50 Table 1:** Clinico-epidemiological features of paediatric Lupus patients

Patient	Gender	Age	End Organ Involvement	Autoantibody profile	Treatment	Current Clinical Status
1	Female	14 years	Arthritis	ANA +ve	Declined treatment of Hydroxychloroquine and Methotrexate to seek 2^nd^ opinion	Lost to follow up
				Antiphospholipid antibody +ve (B2 glycoprotein IgA)		
2.	Female	14 years 9 months	Skin	ANA +ve	Hydroxychloroquine	Lost to follow up
				Anti ds DNA +ve		
3.	Female	11years	Arthritis	ANA+ve >1:640 (fine granular)	Mycophenolate Mofetil	Stable with minimally active disease
			Interstitial Lung Disease	Anti Rib +ve	Hydroxychloroquine	
				Anti P protein +ve	Prednisone	
				Anti SSA +ve	1 dose of Rituximab	
4.	Female	15 years 2 months	Lupus Gangrene	ANA +1:640 (Homogenpous)	Cyclophosphamide	Lost to follow up
				Anti Sm +ve	Mycophenolate Mofetil	
				Anti RNP +ve	Aspirin	
				Anti ds DNA +ve		
				Anti nucleosome+ve		
				Anti ribosome +ve		
5.	Female	8 years 7 months	Neurolupus	ANA +VE 1:640(speckled)	Cyclophosphamide (6 courses)	Minimally Active Disease
			Lupus	Anti Sm +ve	Rituximab (2 courses)	
			Gangrene	Anti RNP +ve	Mycophenolate Mofetil	
				Anti Ribosome +ve	Prednisone	
					Hydroxychloroquine	
6.	Female	6 years 11 months	Arthritis	ANA+VE (1:320 homogeneous)	Mycophenolate Mofetil	Minimally Active Disease
			Skin rash	Anti ds DNA +ve	Hydroxychloroquine	
			Class IV Lupus Nephritis	Anti nucleosome +ve	Prednisone	
7.	Female	14 years 6 months	Class II Lupus Nephritis	ANA VE 1:320	Mycophenolate Mofetil	Remission
				(Homogeneous)	Hydroxychloroquine	

